# Domain Specificity or Generality: Assessing the Chinese Implicit Theories Scale of Six Fundamental Psychological Attributes

**DOI:** 10.3389/fpsyg.2020.00142

**Published:** 2020-02-11

**Authors:** Shimin Zhu, Yanqiong Zhuang, Sing-Hang Cheung

**Affiliations:** ^1^Department of Applied Social Sciences, Hong Kong Polytechnic University, Hong Kong, China; ^2^Department of Sociology, Hong Kong Polytechnic University, Hong Kong, China; ^3^Department of Psychology, The University of Hong Kong, Hong Kong, China

**Keywords:** implicit theories, domains, incremental theories, entity theories, psychological attributes

## Abstract

Implicit theories have been widely studied in different domains; however, it is still debatable whether these theories are domain-specific or domain-general. Using the Implicit Theories Scale (ITS) about six fundamental psychological attributes, i.e., intelligence, personality, cognition, feeling, behavior, and emotion, we examined domain specificity versus generality using a factor analytic approach; in addition, we investigated associations between implicit theories about these domains and related psychological attributes. In four sequential studies, we translated a Chinese version of the ITS (Study 1), tested inter-item correlations within and between subscales (Studies 1–4), and conducted exploratory factor analysis (Studies 2 and 3) and confirmatory factor analysis (Studies 3 and 4). We tested associations between implicit theory domains and coping, resilience, grit, and school performance (Studies 3 and 4). Results showed that the six ITS subscales were independent, while the implicit theories about cognition, feeling, behavior, and emotion shared a common component. The implicit theories of intelligence and personality were independent and did not share a common component. The six domains presented different patterns of association with psychological variables. Overall, our results suggest that implicit theories are both domain-specific and domain-general. Future studies are needed to examine the mechanism underlying the domain specificity and generality of implicit theories.

## Introduction

Beliefs about the changeability of one’s own personal attributes predict important everyday-life measures such as mental wellbeing (Schleider et al., 2015) and learning outcomes (Yeager and Dweck, 2012). Dweck (2013) called these malleability beliefs *implicit theories*. Implicit theories are characterized along a continuum ranging from *entity theories* to *incremental theories*. Entity theorists believe in fixed, unchangeable attributes, while incremental theorists believe in malleable attributes. Incremental theory in a particular domain has been shown to predict better outcomes relevant to the same domain. For instance, individuals who hold an incremental theory about emotion often show a lower level of psychological distress compared with those who hold an entity theory (Kneeland et al., 2016; Schroder et al., 2016). Early studies of implicit theories focused on their domain-specific aspect and assumed independence between implicit theories relating to different domains (Dweck et al., 1995b). However, it is also conceivable that a general cognitive schema or framework can drive a shared component between implicit theories across different domains (Schroder et al., 2016). The current study aimed to examine the extent of domain specificity in implicit theories across six different domains – personality, emotion, feeling, behavior, cognition, and intelligence – using a factor analytic approach.

Personality attributes can be defined as consistent patterns of affect, behavior, and cognition across different spatial and temporal contexts (Wilt and Revelle, 2015; Nuzum et al., 2019). In other words, personality provides an overarching explanation of how one feels, behaves, and thinks. However, it remains unclear whether one’s implicit theories about affect, behavior, and cognition similarly share a general component – possibly through one’s implicit theories about personality. On another level, implicit theories about personality and intelligence may also have a shared component. The extent and nature of the overlap between personality and intelligence have been studied over several decades (Ackerman and Heggestad, 1997; Zeidner and Matthews, 2000). Although implicit theories about personality and intelligence are theoretically independent (Dweck et al., 1995b), they are often correlated in empirical studies (Spinath et al., 2003; Hughes, 2015). If general components of implicit theories exist, they can be better predictors of different psychological measures. In the present study, we addressed these important questions about the general components of implicit theories.

Implicit theories about different domains were originally conceptualized as related but different psychological constructs (Dweck et al., 1995b). Dweck et al. (1995b) developed scales for measuring implicit theories with items asking the respondents to rate whether they thought a person as a whole, one’s intelligence, one’s moral character, or the world could be changed. Three factors emerged from their factor analyses of items for measuring implicit theories about intelligence, morality, and the world across different samples. Interestingly, they proposed that implicit theories about a person as a whole could be conceptually related to other implicit theories about a person’s attributes. Furthermore, implicit person theories were found to be significantly predicted by implicit theories of both intelligence and morality. In subsequent studies (Chiu et al., 1997; Yeager et al., 2011, 2014), implicit theories about personality were often examined, instead of either implicit person theory or implicit morality theory. Are implicit theories of personality like implicit person theories as an overarching general construct, or more like implicit theory of morality being separable from implicit theory about intelligence? We measured implicit theories about additional domains beyond personality and intelligence, and tested different predictions on the factor structure underlying the implicit theory items.

Since the original research by Dweck et al. (1995a), studies have examined implicit theories about different domains, among which affect, behavior, and cognition are fundamentally related to personality (Wilt and Revelle, 2015; Nuzum et al., 2019). Implicit theories about two domains related to affect – emotion (Tamir et al., 2007; De Castella et al., 2015) and feeling (Schleider and Weisz, 2016) – have previously been studied. Some studies define emotions as mental states that arise as a response to external stimuli, and feelings as products involving cognitive evaluations (Damasio, 1995). Some distinguish the two because feelings are necessarily conscious, while emotions are not (Prinz, 2005). Although emotion and feeling are related, the overlap between implicit theories about emotion and feeling is not well understood. With feeling involving a cognitive component, implicit theories about feeling and thoughts may additionally share a common component. Schleider and Weisz (2016) reported strong correlations between implicit theories about feelings, behaviors, and thoughts, ranging from 0.73 to 0.96. Such strong correlations are consistent with the hypothesis that the implicit theories about affect, behavior, and cognition share a general component.

A general component in implicit theories across different domains can offer insights into understanding individuals’ resilience. Many studies have shown that incremental theories are associated with higher resilience and positive coping. For example, an incremental theory about intelligence was associated with higher resilience toward school adversity; similarly, an incremental theory about personality was linked to higher resilience toward peer victimization (Yeager and Dweck, 2012). Individuals who think of a person as more malleable are more resilient in times of hardship (Ng and Tong, 2013; Ryazanov and Christenfeld, 2018), more highly equipped with internalized grit in goal pursuit (Hochanadel and FInamore, 2015; Polirstok, 2017), and better at facing uncertainty during life transitions (Tamir et al., 2007). Incremental theory also predicts better academic performance (Dweck, 2013). In our current study, we sought to examine associations between implicit theories about different domains and attributes related to academic performance and coping with adversity.

The relative contributions from general and domain-specific components to items measuring implicit theories can be examined in factor analysis using *bifactor* models – an approach that has recently re-emerged (Reise, 2012). In a bifactor model, each item can be a result of two underlying latent constructs – one being linked to most, if not all, of the items, and the other being linked to a smaller subset of the items. Here, we hypothesized that a general factor contributed to all the items across implicit theories about different domains, in addition to the contribution from domain-specific factors. Alternatively, a shared component can contribute to the domain-specific factors as a second-order latent construct. In other words, the shared component does not contribute to the items directly, but rather does so indirectly through the domain-specific factors in a *second-order* factor model. Bifactor models have been shown to have advantages over second-order factor models in terms of delineating the underlying factor structure (Chen et al., 2006). The factor structure underlying the items relating to implicit theories about six domains was examined by fitting and comparing bifactor and second-order factor models.

In summary, the objective of the current study was twofold. First, we aimed to test the hypothesis of the existence of a general component shared across implicit theories relating to different domains. Second, we further tested the hypothesis that implicit theories relating to different domains could predict resilience-related variables and academic performance through both the general and the specific components underlying different domain-specific implicit theories. We tested these hypotheses in two studies involving Chinese university students, after validating the Chinese version of the items measuring implicit theories in another two studies of university students.

## A Four-Study Investigation

We undertook this investigation to first translate and validate the Chinese version of the Implicit Theories Scale (ITS) based on six fundamental attributes, i.e., implicit theories of intelligence, personality, cognition, behavior, emotion, and feeling. We further examined the domain specificity of implicit theories on selected fundamental attributes in four studies. In Study 1, we translated the ITS and tested the Chinese translation of the items (*N*_1_ = 183). In Study 2, we examined the average inter-item correlations within and between subscales to establish internal consistency, and used exploratory factor analysis (EFA) to examine the underlying factor structure (*N*_2_ = 146). We also used a bifactor model to test if a general factor could explain all the items. In Study 3, we conducted confirmatory factor analysis (CFA) to test the factor structure of the six implicit theories subscales (*N*_3_ = 355). We also tested the longitudinal invariance of the factor structure. We further assessed the relationship between implicit theories and psychological attributes related to coping with adversity. In Study 4, we identified the relationships between implicit theories and grade point average (GPA) among a sample of Chinese university students (*N*_4_ = 1,731).

Analyses were performed in R Statistical Software (R Development Core Team, 2011). Table 1 outlines the demographic data and study objectives of the four studies. In each study, we report averages within a subscale, and between-subscale inter-item correlations with their range, which is a recommended empirical measure of internal consistency and domain relationship (Clark and Watson, 1995). Ethical approval for the four studies was obtained from the institutional review board of the first author’s university, and all participants (*N*_*total*_ = 2,415) provided their consent to participate in these studies.

**TABLE 1:** Brief descriptions of the four studies.

	Sample size	Age *M* (SD)	Male %	Objectives
Study 1	183	20.6 (0.8)	27.9	Pilot test for translation
Study 2	146	19.8 (1.5)	32.2	EFA
Study 3	355	20.1 (1.0)	18.0	CFA and correlation^#^
Study 4	1,731	20.7 (1.3)	18.8	Examine association with GPA

*EFA, exploratory factor analysis; CFA, confirmatory factor analysis; GPA, grade point average. ^#^Correlations between implicit theories and selected psychological factors were examined.*

### Study 1

#### Purpose

We translated the ITS into Chinese to conduct a pilot test to improve understandability of the Chinese translation.

#### Methods

The Back-Translation and Target Language Test Method was used (Maneesriwongul and Dixon, 2004). This process included six steps: (a) translation by two independent translators; (b) first revision of the translation based on feedbacks from a translation panel; (c) a pilot study among university students (*N* = 183, *M*_*age*_ = 20.6 years, *SD*_*age*_ = 0.8 years, 28% male); (d) second revision based on feedbacks on the understandability of the translated items from two focus groups with 20 participants randomly selected from the pilot sample; (e) back translation to English by two bilingual speakers for comparison with the original scale; and (f) interviews with selected local scholars who have performed studies on implicit theories for their inputs on cultural issues. After collecting all feedbacks from participants and conducting interviews with the expert panel, we made a final revision.

We sent invitations to Year-3 classes randomly selected from one university to recruit participants for Study 1. Students were invited to stay after class to complete the questionnaire. They received a souvenir (with a value of US$3) as compensation for their time. The questionnaire took about 15 min and the focus group took about 20 min.

The first version of the scale consisted of 23 items in six dimensions on a seven-point Likert scale (1 = strongly disagree to 7 = strongly agree). The intelligence domain consisted of three items, and the other domains consisted of four items each. A higher score indicated a stronger belief that the attribute was fixed and unchangeable. Reversed items represented a belief in the changeability and malleability of the attribute. The sample items for the implicit theories were: (a) Intelligence: “You have a certain amount of intelligence and you really cannot do much to change it”; (b) Personality: “Everyone is a certain kind of person and there is not much that can be done to really change that”; (c) Thoughts: “You can change your thoughts if you don’t like them” (Reverse-scoring item); (d) Feelings: “You can control the feelings you have” (Reverse-scoring item); (e) Behavior: “You can change how you behave if you really try” (Reverse-scoring item); and (f) Emotion: “No matter how hard they try, people can’t really change the emotions that they have.”

#### Results and Discussion

Table 2 presents the mean inter-item correlations within and between subscales, indicating the level of internal consistency for the different subscales. The average within-subscale inter-item correlations (*r* = 0.35–0.66) indicated satisfactory internal consistency for the five of the six subscales, except for the emotion dimension (*r* = 0.12; Clark and Watson, 1995; Briggs and Cheek, 1986). We speculated that the low internal consistency for the emotion subscale was due to an order effect. The emotion subscale was located in the first part of the questionnaire, and participants could be still getting used to the question format, which might have influenced the internal consistency of this subscale. Therefore, we adjusted the order of the subscales in subsequent studies. With the new order, items for the intelligence domain would appear first and those for the emotion domain would appear last.

**TABLE 2:** Summary statistics and mean inter-item correlations of implicit theories measures.

Implicit theories domains	Number of item	Likert-scale	*M* (SD)	Mean inter-item correlations (min, max)	
				Within-subscale	Between-subscale
Study 1 (*N* = 183)	23	1–7			
Emotion	4		2.91 (0.72)	0.12 (0.01, 0.35)	0.12 (−0.16, 0.34)
Intelligence	3		3.57 (1.35)	0.66 (0.54, 0.73)	0.18 (−0.04, 0.48)
Personality	4		4.05 (0.95)	0.35 (0.03, 0.65)	0.14 (−0.06, 0.48)
Cognition	4		2.78 (0.85)	0.41 (0.24, 0.50)	0.18 (−0.05, 0.44)
Behaviors	4		2.80 (0.80)	0.39 (0.23, 0.51)	0.13 (−0.16, 0.38)
Feeling	4		3.15 (0.93)	0.42 (0.27, 0.56)	0.19 (−0.04, 0.44)
Study 2 (*N* = 146)	23	1–7			
Intelligence	3		3.55 (1.37)	0.80 (0.76, 0.82)	0.12 (−0.06, 0.26)
Personality	4		3.81 (0.97)	0.38 (0.08, 0.68)	0.15 (−0.06, 0.39)
Cognition	4		2.83 (0.81)	0.42 (0.31, 0.52)	0.24 (−0.04, 0.62)
Behaviors	4		2.86 (0.88)	0.49 (0.30, 0.60)	0.26 (−0.06, 0.62)
Feeling	4		3.17 (0.94)	0.41 (0.26, 0.60)	0.22 (−0.11, 0.58)
Emotion	4		2.83 (0.91)	0.32 (0.08, 0.60)	0.12 (−0.16, 0.34)
Study 3 (*N* = 355)	18	1–6			
Intelligence	3		3.36 (0.96)	0.73 (0.71, 0.75)	0.17 (0.02, 0.32)
Personality	3		4.05 (0.93)	0.66 (0.63, 0.68)	0.12 (−0.03, 0.32)
Cognition	3		2.52 (0.66)	0.48 (0.45, 0.51)	0.23 (0.05, 0.46)
Behaviors	3		2.48 (0.68)	0.49 (0.43, 0.53)	0.18 (0.00, 0.46)
Feeling	3		2.89 (0.81)	0.60 (0.55, 0.67)	0.22 (0.05, 0.43)
Emotion	3		2.48 (0.81)	0.44 (0.24, 0.69)	0.19 (−0.03, 0.43)
Study 4 (*N* = 1,731)	18	1–6			
Intelligence	3		3.44 (0.96)	0.64 (0.60, 0.67)	0.15 (0.07, 0.30)
Personality	3		4.13 (0.91)	0.62 (0.60, 0.65)	0.12 (−0.02, 0.30)
Cognition	3		2.70 (0.73)	0.56 (0.54, 0.57)	0.25 (0.01, 0.51)
Behaviors	3		2.66 (0.71)	0.47 (0.37, 0.54)	0.22 (−0.02, 0.51)
Feeling	3		2.98 (0.88)	0.61 (0.57, 0.69)	0.24 (0.04, 0.45)
Emotion	3		2.53 (0.77)	0.41 (0.28, 0.51)	0.21 (0.06, 0.37)

**M*, mean of the subscale score; SD, standard deviation of the subscale score.*

### Study 2

#### Purpose

Study 2 was a cross-sectional study administering surveys in a classroom setting with three objectives: (a) to re-examine the internal consistency of the subscales after re-ordering the items; (b) to examine the factor structure of the ITS using EFA; and (c) to examine the domain specificity and generality of implicit theories using bifactor EFA models. In bifactor models, the variance in each item was explained by both a general factor and a specific (domain) or group factor. Domain generality would predict a strong general factor explaining all the items across the different domains.

#### Methods

##### Participants and procedure

Participants were 146 university students (*M*_*age*_ = 19.8 years, *SD*_*age*_ = 1.5 years, 32% male), selected randomly from two universities in China. The procedure was similar to that in Study 1, with the only difference being participant recruitment done in two universities instead of one. Informed consent was obtained from each participant. Participants were invited to complete a paper-form questionnaire in a classroom setting after a lecture. This survey took about 15 min to complete.

##### Measures

Participants reported their age, gender, and scores on the ITS, which included 23 items comprising six domains, with a seven-point Likert-type scale ranging from 1 (strongly disagree) to 7 (strongly agree).

##### Analyses

As in Study 1, average within-subscale inter-item correlations were used to examine the internal consistency of each subscale. Further, Velicer’s minimum average partial (MAP) correlation was used to guide the decisions on the number of factors to be retained in EFA (Velicer, 1976). We also reported the empirically estimated Bayesian information criterion (eBIC) as a measure of the goodness of fit for the different factor models. The factor models were estimated with a generalized least squares approach. Factor loadings were rotated with an oblique Crawford–Ferguson criterion. Furthermore, we used two exploratory bifactor analysis methods to the bifactor models, including the Jennrich–Bentler (JB) analytic bifactor rotation and Schmid–Leiman (SL) orthogonalization (Mansolf and Reise, 2016; Irwing et al., 2018). Along with the loadings from the bifactor models, we reported the item-level explained common variance (I-ECV), which summarized the proportion of variance being explained by the general factor for each item.

#### Results and Discussion

The average within-subscale inter-item correlations were moderate to high (*rs* = 0.32–0.80; Table 2), suggesting that all subscales had satisfactory internal consistency (Clark and Watson, 1995). It was worth noting that, in contrast with Study 1, satisfactory internal consistency was also found for the emotion subscale. The between-subscale inter-item correlations (*rs* = 0.12–0.26) were much lower than the within-subscale ones, suggesting that the different subscales were separable from each other. A closer examination revealed that the between-subscale inter-item correlations of cognition, behavior, and feeling (*rs* = 0.22–0.26) were slightly higher than those of intelligence, personality, and emotion (*rs* = 0.12–0.15). Implicit theories of cognition, behavior, and feeling could have lower domain specificity, compared to implicit theories of intelligence, personality, and emotion.

Table 3 presents the MAP and eBIC for factor models with different number of factors (1–6). The eigenvalues for the first six components were 6.67, 2.53, 2.13, 1.63, 1.21, and 1.10. The four-factor model had the lowest MAP. Therefore, we proceeded to extract four factors. Factor loadings from this four-factor model are presented in Table 4. In general, items from implicit theories of cognition and behavior loaded highly on the same factor. Similarly, items from implicit theories of feeling and emotion loaded highly on the same factor. For each of the six original domains, there was one item with loadings of <0.50. We therefore refined the ITS by removing these items with three items remained for each of the six original subscales. We ran EFA again on the remaining 18 items. The eigenvalues for the first six components were 5.46, 2.42, 2.10, 1.58, 0.95, and 0.85. MAP also suggested a four-factor model for these 18 items (Table 3). Factor loadings for these 18 items were also shown in Table 4. For the English and Chinese version of 18-item scale and the correlation table of Study 1 and 2, please refer to Supplementary Materials.

**TABLE 3:** Statistics summary and the mean of the correlation of within-subscale and between-subscale of implicit theories measures.

Number of factors	23-item version		18-item version	
	MAP	eBIC	MAP	eBIC
1	0.031	59	0.046	376
2	0.030	−298	0.042	24
3	0.027	−528	0.039	−267
4	**0.021**	−556	**0.029**	−**357**
5	0.024	−**623**	0.034	−314
6	0.028	−557	0.041	−259

*MAP, Velicer’s Minimum Average Partial; eBIC, empirically estimated Bayesian information criterion. The bolded numbers indicate the smallest values for MAP and eBIC.*

**TABLE 4:** Results of exploratory factor analysis (EFA) of the Implicit Theories Scale from Study 2.

Items	Factor loadings							
	23-item version				18-item version			
	F1	F2	F3	F4	F1	F2	F3	F4
**Intelligence**								
A1. You have a certain amount of intelligence and you really cannot do much to change it.	–0.11	**0.91**	–0.11	0.00	–0.07	**0.91**	–0.05	–0.05
A2. Your intelligence is something about you that you cannot change very much.	–0.04	**0.92**	–0.05	0.03	0.02	**0.93**	0.01	–0.03
A3. You can learn new things, but you can’t really change your basic intelligence.	–0.09	**0.88**	0.03	0.00	–0.01	**0.87**	0.08	–0.04
**Personality**								
A4. The kind of person someone is, something very basic about them, and it can’t be changed very much.	0.02	0.15	**0.72**	–0.03	0.04	0.10	**0.71**	0.01
A5. People can do things differently, but the important parts of who they are can’t really be changed.	–0.03	0.07	**0.84**	–0.18	–0.03	0.03	**0.88**	–0.15
A6. Everyone is a certain kind of person and there is not much that can be done to really change that.	–0.05	–0.01	**0.82**	0.01	–0.06	–0.06	**0.85**	0.06
A7. Everyone is either a winner or a loser in life.	–0.02	–0.04	**0.16**	0.08	−	−	−	−
**Cognition**								
A8. You can control what you think, if you try.	**0.43**	–0.04	0.07	0.21	−	−	−	−
A9. When you don’t like the thoughts you have, you can change them.	**0.60**	0.11	–0.01	0.15	**0.57**	0.09	0.01	0.15
A10. Even if you usually think in a certain way, you can change the thoughts you have.	**0.54**	0.12	0.09	–0.01	**0.48**	0.09	0.11	0.01
A11. You can change your thoughts if you don’t like them.	**0.65**	0.05	0.07	0.11	**0.69**	0.02	0.07	0.10
**Behavior**								
A12. You can change how you behave if you really try.	**0.66**	0.14	0.12	0.12	**0.67**	0.11	0.12	0.11
A13. You can always choose how you behave.	**0.55**	–0.05	0.15	–0.05	**0.56**	–0.07	0.16	–0.04
A14. If you put your mind to it, you can control how you behave.	**0.62**	0.09	0.11	0.04	**0.66**	0.06	0.12	0.03
A15. Even if you usually behave in a certain way, you can change your behavior.	**0.49**	0.15	0.20	0.18	−	−	−	−
**Feeling**								
A16. When you try, you can change the feelings you have.	0.37	0.05	0.19	**0.35**	−	−	−	−
A17. When you feel bad, you can make yourself feel better.	–0.02	0.13	0.05	**0.51**	–0.02	0.13	0.06	**0.48**
A18. You can control the feelings you have.	0.05	0.05	–0.15	**0.64**	0.10	0.04	–0.14	**0.61**
A19. Even if you usually feel a certain way, you can change the feelings you have.	0.03	0.18	0.16	**0.69**	0.08	0.16	0.16	**0.68**
**Emotion**								
A20. Everyone can learn to control their emotions.	0.09	–0.06	0.02	**0.58**	0.09	–0.06	0.00	**0.58**
A21. If they want to, people can change the emotions that they have.	0.12	0.01	0.19	**0.66**	0.12	–0.01	0.18	**0.67**
A22. No matter how hard they try, people can’t really change the emotions that they have.	0.05	0.10	0.19	**0.59**	0.06	0.07	0.18	**0.61**
A23. The truth is, people have very little control over their emotions.	0.08	0.17	0.17	–0.08	−	−	−	−
**Correlations among factors**								
F2	0.17	−			0.13	−		
F3	0.32	0.19	−		0.30	0.17	−	
F4	0.31	0.11	0.19	−	0.31	0.15	0.15	−

*The bolded values highlight the factor loadings yielded from EFA. F1 to F4 were factors derived from EFA, representing the factors Cognition and Behavior (F1); Intelligence (F2); Personality (F3); Feeling and Emotion (F4).*

F1 had moderate inter-factor correlations with F3 and F4 (Table 4) suggesting the possibility of a common factor behind the items of some of these subscales. We examined this possibility by fitting bifactor models with one general factor and four specific factors to the 18-item data set. Both exploratory bifactor analysis approaches, i.e., JB and SL, resulted in similar models. Table 5 presents the factor loadings for the bifactor models estimated using the two approaches. Items from the cognition, behavior, feeling, and emotion domains loaded heavily on the general factor. The JB-based I-ECV indicated that a large proportion of variance in the items of the cognition, behavior, emotion, and feeling domains could be explained by a general factor, while the items from the intelligence and personality domains did not load heavily on the general factor (Table 5).

**TABLE 5:** Results of bifactor analyses of the Implicit Theories Scale from Study 2.

Items	Jennrich–Bentler rotation					Schmid–Leiman orthogonalization					I-ECV
	General	F1	F2	F3	F4	General	F1	F2	F3	F4	
A1	0.15	**0.87**	−0.01	0.06	−0.02	0.18	−0.03	−0.01	**0.87**	−0.04	0.03
A2	0.29	**0.88**	0.02	−0.01	0.00	0.30	0.03	0.01	**0.88**	0.00	0.10
A3	0.27	**0.83**	0.09	0.01	−0.01	0.28	0.01	0.00	**0.82**	0.07	0.09
A4	0.38	0.10	**0.64**	0.05	−0.11	0.42	0.04	0.04	0.08	**0.61**	0.25
A5	0.28	0.06	**0.80**	−0.08	−0.03	0.34	0.02	−0.08	0.03	**0.78**	0.11
A6	0.40	−0.05	**0.73**	−0.01	0.10	0.39	−0.02	0.09	−0.07	**0.74**	0.23
A9	**0.54**	0.01	−0.04	0.02	−0.50	**0.55**	0.35	0.09	0.04	−0.08	**0.53**
A10	**0.42**	0.04	0.09	−0.06	−0.42	**0.46**	0.30	−0.01	0.05	0.04	**0.48**
A11	**0.61**	−0.05	0.01	−0.16	−0.41	**0.62**	0.42	0.04	−0.03	−0.03	**0.66**
A12	**0.66**	0.03	0.04	−0.16	−0.37	**0.66**	0.42	0.06	0.05	0.01	**0.72**
A13	**0.51**	−0.11	0.07	−0.48	−0.05	**0.47**	0.36	−0.07	−0.11	0.08	**0.51**
A14	**0.67**	0.01	0.01	−0.50	−0.08	**0.60**	0.41	−0.01	0.01	0.02	**0.64**
A17	**0.42**	0.07	−0.05	0.31	0.03	**0.29**	−0.05	0.42	0.09	0.00	**0.62**
A18	**0.50**	−0.04	−0.27	0.28	0.06	**0.33**	0.02	0.50	0.00	−0.20	**0.62**
A19	**0.72**	0.07	−0.02	0.32	0.10	**0.53**	0.01	0.59	0.10	0.05	**0.82**
A20	**0.55**	−0.12	−0.17	0.14	0.25	**0.34**	0.01	0.48	−0.10	−0.08	**0.71**
A21	**0.71**	−0.09	−0.01	0.29	0.08	**0.51**	0.03	0.57	−0.06	0.06	**0.83**
A22	**0.63**	0.00	0.01	0.32	0.05	**0.47**	0.00	0.53	0.03	0.07	**0.79**

*I-ECV, item-level explained common variance. I-ECV reported here were calculated based on the Jennrich–Bentler solution of the bifactor model.*

Our EFA results were consistent with domain generality for implicit theories of cognition, behavior, feeling, and emotion. However, the intelligence and personality domains did not share a general factor. They were independent of one another and of the other subscales, which suggested the domain specificity for implicit theories of intelligence and personality. Based on the models identified through EFA, we subsequently conducted Study 3 to further examine the factor structure of ITS using CFA.

### Study 3

#### Purpose

We conducted a two-wave classroom survey among random students from three Chinese universities with three aims: (a) to conduct CFA and test the factor structure of the ITS; (b) to identify its relations with selected psychological attributes; and (c) to examine both longitudinal invariance of the factor structure and test–retest reliability of the scale with the wave-2 data.

#### Methods

##### Participants and procedure

Participants were 355 students (*M*_*age*_ = 20.1 years, *SD*_*age*_ = 1.0 year, 18% male), recruited from Year-2 classes in the three universities. Informed consent was obtained from each participant. Participants were invited to complete a survey in a classroom setting. The survey took about 25 min to complete. After 2 weeks, research assistants administered the wave-2 survey in the same classroom environment. Participants recorded their confidentially safeguarded student number as an identifier for data-matching between the two waves. In addition to using the ITS of fundamental attributes and socio-demographic covariates, we also measured psychological attributes related to adversity coping, including active coping and passive coping, intolerance of uncertainty, dispositional resilience, passion-related grit, and perseverance-related grit.

##### Measures

###### Coping styles

Coping was measured using the 21-item, four-point (0 = “do not do this at all” to 3 = “usually do this a lot”) Chinese Brief COPE Scale (Folkman and Lazarus, 1985; Xie, 1998). Twelve items measured active coping (Cronbach’s alpha in the current study = 0.77). A sample item is “I turn to work or other activities to take my mind off things.” Another nine items measured passive coping styles (Cronbach’s alpha = 0.68). For example, “I use alcohol, tobacco, or other drugs to help me get through it.”

###### Intolerance of uncertainty

Intolerance of uncertainty was measured by the 12-item, five-point (1 = “this is not me” to 5 = “this is very much like me”) Chinese Brief Intolerance of Uncertainty Scale (Carleton et al., 2007; Wu et al., 2016). It consists of a series of statements concerning how people react to various uncertainties in life (Cronbach’s alpha in the current study = 0.85). For example, “I must get away from all uncertain situations” and “I always want to know what the future has in store for me.”

###### Dispositional resilience

Dispositional resilience was measured by the 15-item, three-point (1 = “not at all true” to 3 = “completely true”) Chinese Dispositional Resilience Scale (Bartone, 2007; Tu and Weng, 2013). This scale measures the ability to recover quickly from difficulties or stressful situations. It has three subscales: (a) commitment – “I am full of expectations for my studies/work”; (b) control – “I carefully plan just about everything I do”; and (c) challenge – “I like a lot of change in my work” (overall Cronbach’s alpha in the current study = 0.85).

###### Grit

The power of passion and perseverance was measured by the eight-item, five-point (1 = “very like me” to 5 = “not like me at all”) Grit Scale (Duckworth and Quinn, 2009). The Chinese version was obtained from Duckworth’s laboratory via email. Four items measure passion-related grit (Cronbach’s alpha = 0.75), i.e., how passionate a person is compared to most people. A sample item is “New ideas and projects sometimes distract me from previous ones.” The other four items measure perseverance-related grit (Cronbach’s alpha = 0.69), i.e., how persevering a person is compared to most people. A sample item is “I finish whatever I begin.”

##### Analyses

We conducted a series of CFAs to test the factor structure of the 18-item ITS. We also examined the correlations between ITS and other psychological attributes. Correlations between subscales were also examined. Test–retest reliability, i.e., correlation between the two waves of data, was also examined.

We fitted and compared different CFA models to examine domain specificity and domain generality in ITS. First, we fitted three first-order models (Models 1–3). Model 1 was a one-factor model, which assessed whether domain generality fits the data. Model 2 was a four-factor model, which tested the four factors identified through EFA in Study 2. Model 3 was a six-factor model, which tested whether the six domains were separable factors. Models 4–7 were bifactor models, which tested whether there was a general factor across some domains beyond domain-specific factors. These models, devised according to the results of EFA, were: Model 4 (one general factor with 18 items and 6 group factors); Model 5 (one general factor with 12 items and 4 group factors), Model 6 (one general factor with 15 items, except for the emotion items, and 6 group factors), and Model 7 (one general factor with nine items, including cognition, behavior, and feeling, and six group factors). We also used five second-order models (Models 8–12) to test whether there was a second-order factor across selected domains. These were Model 8 (four factors loaded on a second-order factor: intelligence, personality, cognition and behavior, feeling, and emotion); Model 9 (two factors loaded on a second-order factor: cognition and behavior, and feeling and emotion); Model 10 (all six factors loaded on a second-order factor); Model 11 (four factors loaded on a second-order factor: cognition, behavior, feeling, and emotion); Model 12 (three factors loaded on a second-order factor: cognition, behavior, and feeling). These 12 models were specified according to either the original design of the ITS or different possible models extended from our previous EFA findings in Study 2. Statistics for the CFA models are presented in Table 6.

**TABLE 6:** Results of confirmatory factor analysis (CFA) of the Implicit Theories Scale from Study 3 and Study 4.

	χ^2^	df	χ^2^/df	CFI	TLI	RMSEA	SRMR	MFI	AIC	BIC
**Study 3**										
M1	1,226.79	135	9.09	0.42	0.34	0.18	0.14	0.11	16,438	16,647
M2	352.88	129	2.74	0.89	0.87	0.08	0.06	0.62	15,203	15,435
**M3**	**178.55**	**120**	**1.49**	**0.97**	**0.96**	**0.04**	**0.05**	**0.86**	**14,983**	**15,250**
M4	231.81	117	1.98	0.95	0.93	0.06	0.07	0.78	15,050	15,329
**M5**	**201.22**	**116**	**1.73**	**0.96**	**0.95**	**0.05**	**0.05**	**0.83**	**15,013**	**15,296**
M6	186.86	114	1.64	0.97	0.95	0.05	0.07	0.84	15,003	15,294
M7	15.57	111	1.36	0.98	0.97	0.04	0.05	0.89	14,964	15,266
M8	376.19	131	2.87	0.88	0.86	0.08	0.08	0.60	15,227	15,452
M9	353.37	131	2.70	0.89	0.87	0.08	0.07	0.62	15,200	15,425
M10	227.42	129	1.76	0.95	0.94	0.05	0.08	0.79	15,029	15,261
**M11**	**201.56**	**128**	**1.57**	**0.96**	**0.96**	**0.05**	**0.06**	**0.83**	**14,998**	**15,234**
M12	20.96	127	1.58	0.96	0.96	0.05	0.07	0.83	15,002	15,242
**Study 4**										
M3	419.90	120	3.50	0.97	0.96	0.04	0.03	0.89	75,768	76,145
M5	354.77	116	3.06	0.96	0.95	0.05	0.04	0.84	75,977	76,375
M11	471.04	128	3.68	0.96	0.96	0.05	0.04	0.87	75,820	76,152

*χ^2^, Chi-square; χ^2^/df, Chi-square/degree of freedom; CFI, comparative fit index; TLI, Tucker–Lewis index; RMSEA, root-mean-square error of approximation; SRMR, standardized root mean square residual; MFI, McDonald fit index; AIC, Akaike Information Criterion; BIC, Bayesian Information Criterion. M1, 1-factor model; M2, 4-factor model (as suggested by EFA results); *M3*, *6-factor model*; M4, bifactor model (general factor with 18 items + 4 group factors); *M5*, *bifactor model* (*general factor with 12 items + 4 group factors*); M6, bifactor model (general factor with 15 items except the emotion items + 6 group factors); M7, bifactor model (general factor with nine items including the cognition, behavior, and feeling items + 6 group factors); M8, second-order model (four first-order factors – intelligence, personality, cognition + behavior, and feeling + emotion – loaded on a second-order factor); M9, second-order model (two first-order factors – cognition + behavior and feeling + emotion – loaded on a second-order factor); M10, second-order model (all six factors loaded on a second-order factor); *M11*, *second-order model* (*four first-order factors* – *cognition, behavior, feeling, and emotion* – *loaded on a second-order factor*); M12, second-order model (three first-order factors – cognition, behavior, and feeling – loaded on a second-order factor); M3, M5, and M11 fitted the data well in Study 3, and were fitted to the data from Study 4. M7 fitted well in Study 3, but estimation of M7 did not converge in Study 4. M3 was the best-fitting model, although both M5 and M11 also fitted the data well. The bolded values highlight best-fitting model. The Model 3 (M3) was the best-fit model, while Model 5 (M5) and Model 11 (M11) also fit the data well.*

#### Results and Discussion

Model 3 (the six-factor model) was the best-fit model, while Model 5 (one general factor on 12 items and four group factors) and Model 11 (second-order model, with four factors – cognition, behavior, feeling, and emotion – loaded on a second-order factor) also fit the data well (bold values in Table 5). Model 3 suggests that the six implicit theories domains are empirically distinguishable from each other, and the subscales can be used separately to measure individual domains. The superior model fits in Models 3, 5, and 11 suggested that not all of the six studied domains share a single common factor. Instead, a subset of the domains might share a common factor. Model 5 represented the model identified in our previous EFA where a general component was shared by four domains. Model 11 represented another way of relating a general factor to the items of the four domains, with the first-order factors in between. The results from Model 5 and Model 11 suggested that the implicit theories of cognition, behavior, feeling, and emotion exhibited a domain general feature. Our CFA results revealed that implicit theories relating to these six domains were separable and that implicit theories of cognition, behavior, feeling, and emotion pertained to a general sentiment.

Bivariate correlations between the six studied implicit theories and other adversity coping attributes are reported in Table 7. We used Steiger’s (1980) test, implemented by Lee and Preacher (2013), to examine the differences between correlations. The implicit theories of cognition and feeling had significantly higher correlations with positive coping [*rs* = −0.40 (cognition) and −0.35 (feeling), *ps* < 0.001], resilience (*rs* = −0.53 and −0.40, *p* < 0.001), and perseverance-related grit (*rs* = −0.47 and −0.33, *p* < 0.001) than the correlations between these attributes and implicit theories of intelligence [*rs* = −0.11 (positive coping), −0.29 (resilience), and −0.16 (perseverance-related grit), *ps* < 0.05] and personality [*rs* = −0.15 (positive coping), −0.20 (resilience), and −0.12 (perseverance-related grit), *ps* < 0.05]. The *z*-scores of the differences ranged from 1.61 to 5.09, *ps* < 0.05. We also found that implicit theory of intelligence was positively associated with passion-related grit (*r* = 0.20, *p* < 0.001). Implicit theory of personality was positively associated with intolerance of uncertainty (*r* = 0.26, *p* < 0.001). The passive coping style was not significantly correlated with any implicit theories. These results suggested that different implicit theories had differentiable patterns of correlations with attributes related to adversity coping. The correlation between implicit theories of intelligence and personality was similar to those reported in existing literature (*r* = 0.34, *p* < 0.001) (see Table 8).

**TABLE 7:** Domain-level correlation with relevant psychological attributes in Study 3.

	Intelligence	Personality	Cognition	Behavior	Feeling	Emotion
Positive coping	−0.11*	−0.15**	−0.40***	−0.22***	−0.35***	−0.22***
Negative coping	0.07	0.02	0.07	0.08	–0.03	0.02
Intolerance of uncertainty	0.14**	0.26***	0.16**	–0.02	0.24***	0.12*
Dispositional resilience	−0.29***	−0.20***	−0.53***	−0.38***	−0.40***	−0.27***
Passion-related grit	0.20***	0.17**	0.19***	0.03	0.10	0.13*
Perseverance-related grit	−0.16**	−0.12*	−0.47***	−0.32***	−0.33***	−0.27***

Coping was measured by the Chinese version of Brief Cope Scale, Positive coping, the higher score means more active coping style; and negative coping refer to passive coping style, the higher score means more active coping style or more inactive coping style, respectively; intolerance of uncertainty was measured by the Chinese version of Brief Intolerance of Uncertainty Scale, the higher score means more unable to stand with the uncertainty situation; dispositional resilience was measured by the Chinese version Dispositional Resilience Scale, the higher score means more resilience; passion-related grit and perseverance-related grit were measured by the Chinese version of Grit Scale, the higher score means more passion-related grit or more perseverant respectively. ***p < 0.001; **p < 0.01; *p < 0.05.

**TABLE 8:** Correlation between implicit theories subscales in Study 3 and Study 4.

	alpha (S3)	Intelligence	Personality	Cognition	Behavior	Feeling	Emotion	alpha (S4)
Intelligence	0.89	–	0.32	0.19	0.14	0.17	0.21	0.84
Personality	0.85	0.34	–	0.12	0.08	0.14	0.16	0.83
Cognition	0.74	0.19	0.13	–	0.58	0.52	0.40	0.79
Behavior	0.74	0.18	0.07	0.53	–	0.42	0.39	0.73
Feeling	0.82	0.23	0.18	0.4	0.28	–	0.43	0.82
Emotion	0.71	0.17	0.08	0.44	0.26	0.47	–	0.69

*The correlation and Cronbach’s alpha were calculated based on aggregate scores. The correlation coefficients in the lower diagonal are the correlation of Study 3, and those in the upper diagonal are of Study 4. The *p*-value of all correlation coefficients is <0.05.*

We then readministered the questionnaire after 2 weeks (*N* = 127) and examined the longitudinal invariance of the factor structure. Our results supported a weak invariance model, in which the factor loadings were held constant across the two time points (Chi-square/degree of freedom = 1.26; comparative fit index = 0.93; Tucker–Lewis index = 0.92; root-mean-square error of approximation = 0.05; standardized root-mean-square residua = 0.07; McDonald fit index = 0.32; Akaike information criterion = 10,049; Bayesian information criterion = 10,561). The correlations between two points of the six domains were *r* = 0.56 (intelligence), *r* = 0.56 (personality), *r* = 0.81 (cognition), *r* = 0.67 (behavior), *r* = 0.66 (feeling), and *r* = 0.62 (emotion).

These CFA results corroborate the EFA results from Study 2. While the six domains were indeed separable, some – cognition, behavior, emotion, and feeling – share a common underlying factor. These results may imply that the implicit theory of cognition closely is related to the implicit theory of behavior, and the implicit theory of emotion would be more similar to that of feeling. The reason for this may be related to their perception and categorization of the nature of these domains. Emotion and feeling are both about one’s affect (Stangor et al., 2014). Further, cognitive behavior therapy assumes that there is a strong associative tie between one’s cognition and behavior (Beck, 2011).

Study 3 also found that entity theories were positively related to intolerance of uncertainty and passion-related grit, and negatively related to resilience and perseverance-related grit. These findings may indicate that the belief in the possibility of change is important in predicting resilience and perseverance because, when individuals believe that they can grow and change, they are more likely to be more tolerant of uncertainty, be more resilient, and demonstrate more effort toward achieving their goals. Conversely, if they think that their attributes cannot be changed, they may be less likely to actively cope with challenges, be less resilient, and show less perseverance-related grit. Thus, implicit theories of fundamental attributes appear to be a predictor of adversity coping attributes, which has not been studied before in the extant literature.

### Study 4

#### Purpose

Finally, in Study 4, we replicated the CFAs from Study 3, using a separate sample to reconfirm the CFA models, and employed regression to evaluate the specificity of each implicit theory predicting GPA among a sample of consented university students.

#### Methods

##### Participants and procedure

Participants were 1,731 randomly selected university students (*M*_*age*_ = 20.7 years, *SD*_*age*_ = 1.3 years). About 19% of the participants were male – a reasonable representative percentage of the initial population, of which 30% were male. They were recruited via social media invitations sent out by university teachers. The survey took about 15 min to complete. We included five items to assess careless responding (e.g., “please answer Choice 2 to ensure you are paying attention”) and excluded those participants who failed to answer all these items correctly (Schroder et al., 2016).

##### Measures

We assessed the ITS, selected socio-demographic measures, and self-reported GPAs from the previous academic year (2017).

##### Analyses

We replicated the previous CFA findings of Models 3, 5, and 11 from Study 3 to further reconfirm the previously identified factor structure. Linear regression analyses were used to identify the domain-level associations between the implicit theories and GPA of these participants.

#### Results and Discussion

Confirmatory factor analysis was conducted to estimate model fit in Study 4, which echoed those found in Study 3 (Table 6). Model 3, Model 5, and Model 11 recorded the best model fits. Linear regression results showed that gender, age, and implicit theories of cognition were associated with GPA. Being female and being older meant that participants were more likely to have obtained higher GPA. We found that a higher GPA was associated with incremental theories of cognition, i.e., the more one believes that one’s thoughts can change, the more likely one is to have a higher GPA (see Table 9).

**TABLE 9:** Linear regression analysis of GPA with implicit theories variables as predictors among university students in Study 4.

	*b*	SE	β	*t*	VIF
Gender (male)	0.61	0.06	0.23	10.01***	1.03
Age	–0.19	0.02	–0.25	−10.90***	1.01
Intelligence	–0.01	0.03	–0.01	–0.45	1.16
Personality	0.02	0.03	0.02	0.73	1.13
Cognition	0.12	0.04	0.08	2.68**	1.80
Behavior	0.06	0.04	0.04	1.34	1.61
Feeling	–0.01	0.03	–0.01	–0.38	1.52
Emotion	–0.01	0.04	–0.01	–0.41	1.40

GPA, grade point average; VIF, variance inflation factor; *R*^2^ = 0.1239. VIF was smaller than 2 for all predictors suggesting the absence of multicollinearity issues. ***p < 0.001; **p < 0.01.

Among the six main domains, only implicit theories of cognition, rather than intelligence, were found to be associated with GPA. The existing literature suggests that implicit theories of intelligence are associated with the academic performance of secondary school students (Blackwell et al., 2007). However, for the university students in our study, implicit theories of intelligence may not have played such an important role as they did for younger samples of secondary school students. Believing that one’s thought can change, having an open mind, and being ready to change and acquire new knowledge may be more important for university students’ academic performance. Also, this cross-sectional survey may not be able to confirm the causal relationships found between implicit theories and GPA. Since we measured only GPA scores from the last semester, there may be an alternative explanation for the results, in which academic performance can predict implicit theories (Gonida et al., 2006). Future studies should examine the longitudinal interactions between implicit theories and academic performance, along with possible cultural influences, which may contextualize these uncertain and mixed findings.

## General Discussion

People may hold specific and relatively independent beliefs or a relatively global belief that all of one’s attributes are either malleable or fixed. The extent of domain specificity relating to implicit theories in six different domains was studied using four samples and robust factor analysis. We translated and validated the measures of implicit theories of six fundamental attributes and examined the specificity and generality of these domains. We examined average inter-item correlations (Studies 1–4), EFAs (Study 2), and CFAs (Studies 3–4), and tested the psychometric characteristics and factor structure of the ITS. Our findings indicate that the implicit theories of fundamental attributes are distinguishable from one another. There appears to be an underlying general factor that cuts across the domains of cognition, behavior, feeling, and emotion, while the domains of intelligence and personality are relatively independent of the general factor. In other words, if an individual thinks one of the four domains can change, s/he is likely to believe that the other three domains are malleable. However, the belief in change relating to intelligence and personality was independent of the general tendency noted.

Further, implicit theories about one attribute may be related to whether people perceive this attribute as being influenced more by trait than by situation. The assumption is that traits are enduring qualities and are stable over time and situations (Allport, 1937; Corr and Matthews, 2012); thus, intelligence and personality, for instance, may be more likely to be perceived as trait-related fundamental attributes (Ackerman and Heggestad, 1997). For example, if individuals posit intelligence and personality as traits, they would have underpinning independent theories regarding each trait, and they may be more likely to have higher entity beliefs about these attributes, which are usually thought to be more stable and fixed. This was reflected in the mean scores of implicit theories for intelligence and personality, which were higher than those for the other four domains across our four studies (Table 2). Conversely, cognition, behavior, feeling, and emotion are often referred to as basic psychological processes, which are likely to be more state-related attributes (Dolan, 2002). Since state-related attributes are usually perceived as being more situational and concrete (Fridhandler, 1986), individuals may believe that these attributes are changeable, and that such change is subject to culture, situation, context, or the interaction of all of these together. Thus, the general factor overarching implicit theories relating to the four domains and the domain-specific implicit theories relating to intelligence and personality may, in turn, reflect the way people perceive trait–state distinctions relating to these attributes. Further studies are needed to explain the possible underlying dynamic mechanism of domain generality and domain specificity of implicit theories.

The domain specificity and generality of implicit theories may also relate to the perceived closeness of the conceptualization of the attributes. For example, as stated in the section “Introduction,” feelings and emotions related and share some overlap in their concept, with emotion is defined as any of the particular feelings that characterize such a state of mind, and feelings are defined as the emotional side of someone’s character; emotional responses or tendencies to respond (Damasio, 1995). The relatedness of two attributes contributes to the high correlation between implicit theories of the two attributes.

The ITS had satisfactory psychometric properties in our studies. The internal consistency was satisfactory, even though we chose only three items to represent one attribute. Rather than using a single directional of measure of entity theories, we used both directions, i.e., included questions about entity theories and incremental theories, to avoid fatigue in filling in the questionnaire. The mixed combination also had satisfactory internal consistency. CFA revealed the six-factor model to fit best, which indicated that the subscales are relatively independent. Thus, this study provided a usable scale of implicit theories with six subscales that can be employed for measuring different domains of implicit theories in future studies among Chinese samples.

### Limitations and Future Studies

We need to acknowledge certain limitations of this investigation. First, the study only examined domain specificity and generality among implicit theories of six fundamental attributes. Since there are implicit theories of various attributes, the six domains represented only a portion of the implicit theories, and the results may not be the most conclusive and/or comprehensive for a better understanding of the nuanced issues of implicit theories. Also, the six subscales were extracted from different implicit theories studies (Dweck et al., 1995a; Schleider and Weisz, 2016; Schroder et al., 2016). These validated subscales were proved to be reliable for measuring the implicit theories, but variance might be caused due to the different language expressions. Future research into implicit theories should explore more fundamental attributes with a standardized format of measures to further examine the underlying mechanisms of implicit theories. Despite this limitation, this study provided evidence of six of the most representative fundamental attributes, which were previously looked at in individual studies. Although some literature has examined the domain specificity and generality of implicit theories and mental health mind-sets (Schroder et al., 2016) or domain specificity of implicit theories of programming (Scott and Ghinea, 2014), this study has extended the empirical evidence to fundamental attributes, and contributes to furthering the discourse around understanding the domain specificity and generality of implicit theories.

What is more, participants in these four studies were largely female, possibly further limiting the generalizability of the factor structure findings of the implicit theories measures. However, this possible bias is reduced as the gender distribution is merely representative of the gender distribution of the population of the selected universities. Finally, due to the limited time and resources for data collection, we did not include a cultural comparison, which may limit the generalizability of our findings to Western society. Such a cultural comparison would be helpful to integrate these findings in the context of a cultural framework. Nevertheless, this investigation has filled a distinct gap in research by testing the domain specificity and generality of implicit theories among Chinese university students, and provides a reliable and validated Chinese scale for future study among Chinese populations.

## Conclusion

In sum, this investigation presents and tests a Chinese ITS of six fundamental attributes. EFA and CFA indicated that implicit theories of fundamental attributes are unique and distinguishable from one another. There was an underlying general factor that cut across the domains of cognition, behavior, feeling, and emotion. The implicit theories of intelligence and personality were independent of one another, and also of the general factor. These results contribute to the ongoing discourse that aims to better understand the domain specificity and generality of implicit theories, and provide a reliable and validated Chinese scale of implicit theories of six fundamental attributes. Further study and research are warranted and needed in this area as a better understanding of the factors relating to our traits and attributes contributes to a better understanding of how we perceive ourselves both today and in the future.

## Data Availability Statement

The datasets generated for this study are available on request to the corresponding author.

## Ethics Statement

The studies involving human participants were reviewed and approved by the Human Subject Subcommittee of the Hong Kong Polytechnic University. We declare that the ethical standards were in line with the 1964 Helsinki declaration and its later amendments or comparable ethical standards.

## Author Contributions

SZ was responsible for the conception of the research question, study design, and data collection, and drafted the manuscript in consultation with S-HC. YZ assisted with the study design and data collection, and was responsible for data input and cleaning. S-HC was responsible for data analysis and provided feedback regarding the study design. All authors have approved the final version of the manuscript.

## Conflict of Interest

The authors declare that the research was conducted in the absence of any commercial or financial relationships that could be construed as a potential conflict of interest.
