# Supplementary material for: Domain Specificity or Generality: Assessing the Chinese Implicit Theories Scale of Six Fundamental Psychological Attributes
Source: Front Psychol. 2020 Feb 11;11:142. doi: 10.3389/fpsyg.2020.00142 (PMC7027355; doi:10.3389/fpsyg.2020.00142)
Supplement: Supplementary file 1 [file Table_1.DOCX]

Appendix 1. The Chinese Implicit Theories Scale

|  |  | 完全  反對 | 反對 | 有點  反對 | 有點  同意 | 同意 | 完全  同意 |
| --- | --- | --- | --- | --- | --- | --- | --- |
| 1. | 你有一定程度的智力但是你卻無法改變它。 | • | • | • | • | • | • |
| 2. | 你的智力是你的一部分而你無法改變它。 | • | • | • | • | • | • |
| 3. | 你有學習新事物的能力,但是你卻很難改變你本來的智力。 | • | • | • | • | • | • |
| 4. | 每個人的性格都是他們非常基本的一部分並且人們很難改變自己的性格。 | • | • | • | • | • | • |
| 5. | 每個人都可以有不同的做事方式,但是他們很難改變他們自己本身（性格）最重要的部分。 | • | • | • | • | • | • |
| 6. | 每個人都有著特定的人格並且他們都很難改變。 | • | • | • | • | • | • |
| 7. | 當你覺得你的思想不妥當,你能調整它們。 | • | • | • | • | • | • |
| 8. | 即使你常用一種方法去想問題,你仍然能調整它。 | • | • | • | • | • | • |
| 9. | 如果你不滿意自己的想法,你可以改變它們。 | • | • | • | • | • | • |
| 10. | 如果你努力嘗試,你能夠改變你的行為方式。 | • | • | • | • | • | • |
| 11. | 你總是可以選擇你如何表現你的行為舉止。 | • | • | • | • | • | • |
| 12. | 假如你堅持，你可以控制自己如何表現。 | • | • | • | • | • | • |
| 13. | 當你情緒低落時,你能使自己好受一點。 | • | • | • | • | • | • |
| 14. | 你能控制自己的情緒。 | • | • | • | • | • | • |
| 15. | 即使你經常會有某種特定情緒,你仍然能轉變這種心情。 | • | • | • | • | • | • |
| 16. | 每個人都可以學會控制自己的感受。 | • | • | • | • | • | • |
| 17. | 假如人們願意，他們可以改變自己的情緒。 | • | • | • | • | • | • |
| 18. | 無論人們多努力，他們都改變不了自己的情緒。 | • | • | • | • | • | • |

Appendix 2. The Implicit Theories Scale

|  |  | strongly disagree | disagree | a little disagree | a little agree | agree | strongly agree |
| --- | --- | --- | --- | --- | --- | --- | --- |
| 1. | You have a certain amount of intelligence and you really cannot do much to change it. | • | • | • | • | • | • |
| 2. | Your intelligence is something about you that you cannot change very much. | • | • | • | • | • | • |
| 3. | You can learn new things, but you can’t really change your basic intelligence. | • | • | • | • | • | • |
| 4. | The kind of person someone is, something very basic about them and it can’t be changed very much. | • | • | • | • | • | • |
| 5. | People can do things differently, but the important parts of who they are can’t really be changed. | • | • | • | • | • | • |
| 6. | Everyone is a certain kind of person and there is not much that can be done to really change that. | • | • | • | • | • | • |
| 7. | When you don’t like the thoughts you have, you can change them. | • | • | • | • | • | • |
| 8. | Even if you usually think in a certain way, you can change the thoughts you have. | • | • | • | • | • | • |
| 9. | You can change your thoughts if you don’t like them. | • | • | • | • | • | • |
| 10. | You can change how you behave if you really try. | • | • | • | • | • | • |
| 11. | You can always choose how you behave. | • | • | • | • | • | • |
| 12. | If you put your mind to it, you can control how you behave. | • | • | • | • | • | • |
| 13. | When you feel bad, you can make yourself feel better. | • | • | • | • | • | • |
| 14. | You can control the feelings you have. | • | • | • | • | • | • |
| 15. | Even if you usually feel a certain way, you can change the feelings you have. | • | • | • | • | • | • |
| 16. | Everyone can learn to control their emotions. | • | • | • | • | • | • |
| 17. | If they want to, people can change the emotions that they have. | • | • | • | • | • | • |
| 18. | No matter how hard they try, people can’t really change the emotions that they have. | • | • | • | • | • | • |
