# Supplementary material for: Domain Specificity or Generality: Assessing the Chinese Implicit Theories Scale of Six Fundamental Psychological Attributes
Source: Front Psychol. 2020 Feb 11;11:142. doi: 10.3389/fpsyg.2020.00142 (PMC7027355; doi:10.3389/fpsyg.2020.00142)
Supplement: Supplementary file 2 [file Table_2.DOCX]

Supplementary Table: Mean, Standard Deviation, and inter-correlation between items of Study1 and Study 2

|  | M (S1) | SD (S1) | A1 | A2 | A3 | A4 | A5 | A6 | A7 | A8 | A9 | A10 | A11 | A12 | A13 | A14 | A15 | A16 | A17 | A18 | A19 | A20 | A21 | A22 | A23 | M (S2) | SD (S2) |
| --- | --- | --- | --- | --- | --- | --- | --- | --- | --- | --- | --- | --- | --- | --- | --- | --- | --- | --- | --- | --- | --- | --- | --- | --- | --- | --- | --- |
| A1 | 3.56 | 1.50 | - | .82 | .76 | .12 | .07 | .02 | -.03 | 0 | .09 | .14 | .06 | .10 | -.06 | .09 | .17 | .09 | .12 | .04 | .21 | -.03 | .07 | .13 | .17 | 3.60 | 1.44 |
| A2 | 3.36 | 1.51 | .73 | - | .81 | .22 | .14 | .10 | -.01 | .07 | .18 | .11 | .13 | .26 | .06 | .19 | .24 | .18 | .20 | .08 | .24 | .05 | .13 | .18 | .13 | 3.45 | 1.48 |
| A3 | 3.81 | 1.61 | .54 | .70 | - | .25 | .22 | .12 | -.02 | -.01 | .17 | .17 | .11 | .20 | .06 | .17 | .18 | .10 | .16 | .10 | .25 | .05 | .10 | .16 | .20 | 3.61 | 1.51 |
| A4 | 4.44 | 1.51 | .40 | .36 | .41 | - | .63 | .59 | .16 | .13 | .22 | .23 | .27 | .35 | .20 | .25 | .39 | .26 | .20 | .04 | .25 | .05 | .24 | .26 | .21 | 3.99 | 1.53 |
| A5 | 5.10 | 1.30 | .41 | .41 | .48 | .65 | - | .68 | .11 | .15 | .16 | .23 | .14 | .22 | .26 | .24 | .23 | .28 | .04 | -.06 | .18 | -.02 | .13 | .15 | .19 | 4.86 | 1.33 |
| A6 | 4.80 | 1.28 | .30 | .31 | .35 | .54 | .65 | - | .08 | .20 | .12 | .19 | .26 | .23 | .25 | .27 | .32 | .28 | .11 | -.04 | .30 | .13 | .29 | .28 | .11 | 4.54 | 1.31 |
| A7 | 1.85 | 1.08 | .20 | .16 | .15 | .16 | .09 | .03 | - | -.04 | .14 | -.01 | .09 | .17 | -.03 | .02 | .08 | -.01 | -.02 | -.01 | .12 | .09 | .14 | .14 | .07 | 1.84 | 0.97 |
| A8 | 2.71 | 1.27 | .14 | .18 | .22 | .04 | .08 | -.05 | .19 | - | .47 | .32 | .31 | .36 | .27 | .26 | .39 | .42 | .26 | .11 | .20 | .34 | .38 | .28 | .11 | 2.92 | 1.18 |
| A9 | 2.52 | 1.03 | .31 | .21 | .25 | .09 | .11 | .01 | .19 | .50 | - | .49 | .52 | .52 | .31 | .37 | .41 | .39 | .19 | .32 | .30 | .19 | .35 | .34 | .19 | 2.58 | 1.02 |
| A10 | 3.14 | 1.15 | .27 | .21 | .25 | .16 | .15 | -.04 | .12 | .31 | .48 | - | .39 | .43 | .25 | .35 | .49 | .37 | .07 | .12 | .29 | .11 | .30 | .16 | .19 | 3.09 | 1.12 |
| A11 | 2.75 | 1.13 | .14 | .10 | .14 | .15 | .15 | .09 | .02 | .24 | .43 | .47 | - | .62 | .42 | .53 | .49 | .45 | .23 | .22 | .37 | .18 | .29 | .35 | .07 | 2.72 | 1.01 |
| A12 | 2.37 | 0.93 | .09 | .16 | .19 | .10 | .14 | .02 | .19 | .17 | .33 | .32 | .38 | - | .40 | .56 | .60 | .49 | .23 | .17 | .39 | .26 | .42 | .36 | .15 | 2.56 | 1.08 |
| A13 | 3.33 | 1.23 | .02 | -.02 | .05 | .07 | .07 | -.03 | .04 | .01 | .16 | .24 | .18 | .23 | - | .59 | .30 | .31 | .09 | .11 | .17 | .20 | .26 | .14 | .06 | 3.34 | 1.28 |
| A14 | 2.73 | 1.04 | .05 | -.01 | .04 | .13 | .11 | .04 | .10 | -.01 | .16 | .22 | .32 | .38 | .46 | - | .47 | .40 | .10 | .24 | .34 | .27 | .27 | .27 | .05 | 2.71 | 1.04 |
| A15 | 2.77 | 1.13 | .04 | .13 | .08 | .05 | .08 | -.02 | .10 | .04 | .11 | .3 | .26 | .49 | .30 | .51 | - | .46 | .26 | .17 | .41 | .26 | .40 | .39 | .09 | 2.82 | 1.09 |
| A16 | 3.29 | 1.37 | .19 | .13 | .15 | .09 | .11 | -.02 | -.04 | .28 | .24 | .31 | .27 | .29 | .15 | .27 | .37 | - | .37 | .26 | .47 | .29 | .52 | .40 | .06 | 3.28 | 1.25 |
| A17 | 2.93 | 1.28 | .30 | .15 | .09 | .12 | .14 | .07 | .19 | .20 | .41 | .24 | .16 | .08 | -.02 | 0 | .11 | .27 | - | .37 | .39 | .20 | .36 | .41 | .02 | 2.99 | 1.41 |
| A18 | 3.22 | 1.18 | .32 | .19 | .16 | .20 | .25 | .13 | .14 | .28 | .44 | .30 | .37 | .30 | .12 | .34 | .27 | .32 | .55 | - | .60 | .35 | .34 | .34 | -.11 | 3.05 | 1.15 |
| A19 | 3.17 | 1.14 | .34 | .26 | .24 | .13 | .16 | .06 | .11 | .28 | .35 | .39 | .27 | .16 | .20 | .19 | .26 | .30 | .49 | .56 | - | .43 | .58 | .49 | -.03 | 3.34 | 1.25 |
| A20 | 2.31 | 1.32 | -.02 | -.04 | -.04 | .03 | .09 | .02 | .03 | .01 | .09 | .21 | .15 | .24 | .09 | .25 | .11 | .12 | .07 | .15 | .00 | - | .55 | .42 | .08 | 2.47 | 1.37 |
| A21 | 2.60 | 1.27 | .03 | .03 | .07 | .09 | .17 | .09 | -.06 | .23 | .21 | .20 | .31 | .23 | .05 | .26 | .18 | .22 | .09 | .31 | .28 | .17 | - | .60 | .12 | 2.77 | 1.33 |
| A22 | 2.26 | 1.05 | .20 | .18 | .21 | .30 | .25 | .23 | .34 | .16 | .13 | .12 | .10 | .16 | .02 | .09 | .12 | .02 | .12 | .12 | .16 | .03 | .35 | - | .15 | 2.12 | 1.08 |
| A23 | 4.48 | 1.35 | .23 | .10 | .01 | .16 | .17 | .18 | .11 | -.04 | .04 | .05 | .00 | -.01 | -.16 | -.01 | -.05 | .05 | .14 | .22 | .03 | .01 | .03 | .12 | - | 3.97 | 1.44 |

Notes. the correlation coefficients (*r*) between items of Study 1 are below the diagonal and *r*s of Study 2 are above the diagonal.
